# Laparoscopic versus open gastrectomy for gastric cancer, a multicenter prospectively randomized controlled trial (LOGICA-trial)

**DOI:** 10.1186/s12885-015-1551-z

**Published:** 2015-07-29

**Authors:** Leonie Haverkamp, Hylke JF Brenkman, Maarten FJ Seesing, Suzanne S Gisbertz, Mark I van Berge Henegouwen, Misha DP Luyer, Grard AP Nieuwenhuijzen, Bas PL Wijnhoven, Jan JB van Lanschot, Wobbe O de Steur, Henk H Hartgrink, Jan HMB Stoot, Karel WE Hulsewé, Ernst J Spillenaar Bilgen, Jeroen E Rütter, Ewout A Kouwenhoven, Marc J van Det, Donald L van der Peet, Freek Daams, Werner A Draaisma, Ivo AMJ Broeders, Henk F van Stel, Miangela M Lacle, Jelle P Ruurda, Richard van Hillegersberg, LOGICA study group

**Affiliations:** 1 University Medical Center Utrecht, Heidelberglaan 100, 3584 CX Utrecht, The Netherlands; 2 Academic Medical Center, Meibergdreef 9, 1105 AZ Amsterdam, The Netherlands; 3 Catharina Hospital, Michelangelolaan 2, 5623 EJ Eindhoven, The Netherlands; 4 Erasmus Medical Center, ’s-Gravendijkwal 230, 3015 CE Rotterdam, The Netherlands; 5 Leiden University Medical Center, Albinusdreef 2, 2333 ZA Leiden, The Netherlands; 6 Zuyderland Medical Center, Dr. H. van der Hoffplein 1, 6162 BG Sittard-Geleen, The Netherlands; 7 Rijnstate Hospital, Wagnerlaan 55, 6815AD Arnhem, The Netherlands; 8 ZGT Hospitals, location Almelo, Zilvermeeuw 1, 7609 PP Almelo, The Netherlands; 9 VU University Medical Center, De Boelelaan 1117, 1081 HZ Amsterdam, The Netherlands; 10 Meander Medical Center, Maatweg 3, 3813 TZ Amersfoort, The Netherlands

**Keywords:** Gastric cancer, Gastrectomy, Laparoscopy

## Abstract

**Background:**

For gastric cancer patients, surgical resection with *en-bloc* lymphadenectomy is the cornerstone of curative treatment. Open gastrectomy has long been the preferred surgical approach worldwide. However, this procedure is associated with considerable morbidity. Several meta-analyses have shown an advantage in short-term outcomes of laparoscopic gastrectomy compared to open procedures, with similar oncologic outcomes. However, it remains unclear whether the results of these Asian studies can be extrapolated to the Western population. In this trial from the Netherlands, patients with resectable gastric cancer will be randomized to laparoscopic or open gastrectomy.

**Methods:**

The study is a non-blinded, multicenter, prospectively randomized controlled superiority trial. Patients (≥18 years) with histologically proven, surgically resectable (cT1-4a, N0-3b, M0) gastric adenocarcinoma and European Clinical Oncology Group performance status 0, 1 or 2 are eligible to participate in the study after obtaining informed consent. Patients (n = 210) will be included in one of the ten participating Dutch centers and are randomized to either laparoscopic or open gastrectomy. The primary outcome is postoperative hospital stay (days). Secondary outcome parameters include postoperative morbidity and mortality, oncologic outcomes, readmissions, quality of life and cost-effectiveness.

**Discussion:**

In this randomized controlled trial laparoscopic and open gastrectomy are compared in patients with resectable gastric cancer. It is expected that laparoscopic gastrectomy will result in a faster recovery of the patient and a shorter hospital stay. Secondly, it is expected that laparoscopic gastrectomy will be associated with a lower postoperative morbidity, less readmissions, higher cost-effectiveness, better postoperative quality of life, but with similar mortality and oncologic outcomes, compared to open gastrectomy. The study started on 1 December 2014. Inclusion and follow-up will take 3 and 5 years respectively. Short-term results will be analyzed and published after discharge of the last randomized patient.

**Trial registration:**

NCT02248519

## Background

Gastric cancer is the fifth most prevalent cancer and the third most common cause of cancer related death worldwide [1]. Surgical resection with *en-bloc* lymphadenectomy is the cornerstone of curative treatment, however only half of the patients are eligible for surgery with curative intent. The 5-year survival rate after curative resection is 45 % [2]. Perioperative chemotherapy improves 5-year survival with approximately 10 % [3, 4].

Open gastrectomy is the preferred surgical approach worldwide [5]. However, this procedure is associated with considerable morbidity [6–8]. Minimally invasive gastrectomy was introduced in 1993 and aimed at reducing surgical trauma and as a consequence lowering morbidity and mortality [9]. Several systematic reviews and meta-analyses have shown an advantage in short-term outcomes of laparoscopic distal and total gastrectomy compared to open procedures. Oncologic outcomes are similar on the short term [7, 8, 10–12]. However, these studies are mainly performed in the Asian population in which early gastric cancer is detected at a higher rate due to a screening program. This is in contrast to the Western population in which gastric carcinoma is usually diagnosed at an advanced stage [13]. Furthermore, the Western patients are older and have a different spectrum of comorbidities compared to the Asian population [14]. Therefore, it remains unclear whether the results of these Asian studies can be extrapolated to the Western population.

This protocol describes a multicenter, prospectively, randomized controlled trial comparing laparoscopic versus open gastrectomy for gastric cancer in the Netherlands, entitled Laparoscopic versus Open Gastrectomy for gastrIc CAncer (LOGICA-trial).

### Aim of the study

The aim of this multicenter, prospectively randomized controlled superiority trial is to compare laparoscopic gastrectomy versus open gastrectomy in patients with resectable gastric adenocarcinoma. The primary outcome parameter is postoperative hospital stay. Secondary outcome parameters are postoperative morbidity and mortality, oncologic outcomes, readmissions, quality of life and cost-effectiveness.

## Methods

### Objectives

The objective of this study is to compare laparoscopic versus open gastrectomy in patients with resectable gastric adenocarcinoma by means of a randomized controlled trial. The primary outcome parameter is postoperative hospital stay in days. Secondary outcome parameters are postoperative morbidity and mortality, oncologic outcomes, readmissions, quality of life and cost-effectiveness. It is hypothesized that laparoscopic gastrectomy leads to shorter hospital stay, lower postoperative morbidity, less readmissions, higher cost-effectiveness, higher postoperative quality of life, and more patients fit for postoperative chemotherapy, with similar mortality and oncologic outcomes compared to the current standard of care, *i.e.* open gastrectomy.

### Study design

This is a non-blinded multicenter prospectively randomized controlled, superiority trial comparing laparoscopic versus open gastrectomy in patients with resectable gastric adenocarcinoma. This study is conducted in accordance with the principles of the Declaration of Helsinki and Good Clinical Practice Guidelines. The independent ethics committee of the University Medical Center Utrecht (UMC Utrecht) has approved this study for all participating sites. Written informed consent will be obtained from all participating patients. Clinical trial monitoring will be conducted by an independent monitor. A Data Safety Monitoring Board (DSMB) is appointed to evaluate the trial by interim analysis.

### Study population

Patients (≥18 years) with histologically proven, surgically resectable (cT1-4a, N0-3b, M0) gastric adenocarcinoma are eligible to participate in the study. Patients should have performance status 0, 1 or 2 according to the European Clinical Oncology Group (ECOG). Written informed consent is required.

Patients’ inclusion and exclusion criteria are defined as follows:

Inclusion criteria:

- Histologically proven adenocarcinoma of the stomach
- Surgically resectable (cT1-4a, N0-3b, M0) tumor
- Age ≥ 18 years
- ECOG performance status 0,1 or 2.
- Written informed consent

Exclusion criteria

- Siewert type I tumor (tumor located between 1 and 5 cm proximal from the esophago-gastric junction)
- Pregnancy

### Study protocol

Patients will be informed and included at the surgical outpatient department at one of the following ten Dutch investigational centers: University Medical Center Utrecht, Utrecht; Academic Medical Center, Amsterdam; Catharina Hospital, Eindhoven; Erasmus Medical Center, Rotterdam; Leiden University Medical Center, Leiden; Zuyderland MC, Sittard; Rijnstate Hospital, Arnhem; VU University Medical Center, Amsterdam; ZGT Hospital, Almelo; Meander Medical Center, Amersfoort.

The performance status (ECOG) of the patients is assessed. The Dutch guideline on gastric cancer will be used to guide preoperative diagnostic measurements [15]. According to this guideline, all included patients will undergo gastro-esophagoscopy with biopsy and computed tomography of the thorax and abdomen to identify metastatic disease and the extension of the disease before inclusion.

**Fig. 1:**
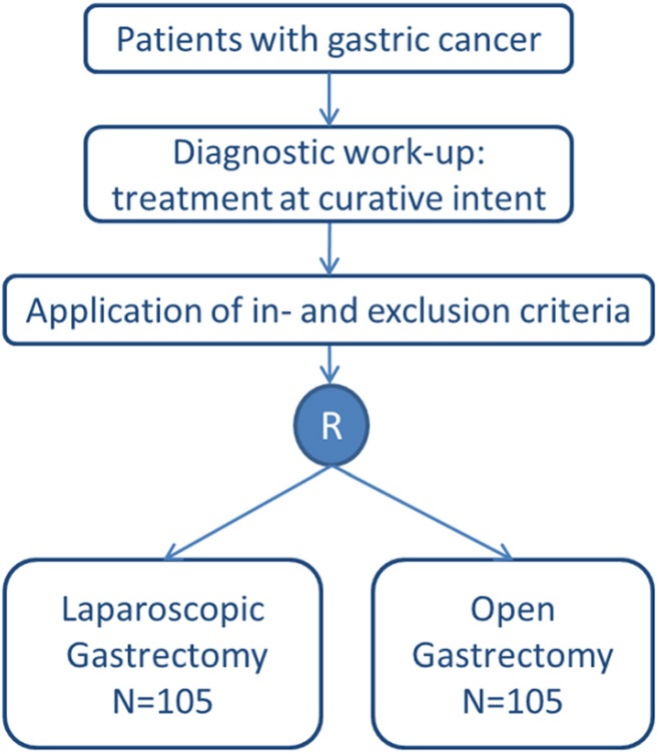
LOGICA-trial flowchart

Patients will receive perioperative chemotherapy according to current Dutch guidelines for gastric cancer [15]. After signing informed consent, the study coordinator will directly randomize participants by means of an online random treatment generator (Fig. 1), stratified by center and type of resection (distal or total gastrectomy). The surgeon, patient and coordinating researcher are not blinded for the allocated treatment. However, the data-analyst will be blinded for the allocated procedure (Fig. 1).

Blood samples will be obtained before surgery, directly postoperatively and on postoperative day two to monitor CRP and leukocyte count to obtain an indication of early inflammatory response after surgery.

The study started on 1 December 2014. Inclusion and follow-up will take 3 and 5 years respectively. The total duration of the study will be 8 years. Study participants can leave the study at any time, for any reason, without any consequences. Study participants will be replaced by newly recruited and randomized subjects in case of withdrawal before surgery. Analysis will be on an intention to treat basis.

### Total gastrectomy

The patient is positioned in supine position under general anesthesia. The conventional open total gastrectomy is performed by means of an upper midline laparotomy. In case of the laparoscopic procedure, the number and placement of the camera, working and assistance ports will be performed according to the surgeons’ preference. After establishment of pneumoperitoneum and introduction of the camera port, the working ports and assistance ports are introduced under direct vision.

In both procedures, first the lesser omentum is divided. Next, the lesser and greater curvatures of the stomach are dissected together with the locoregional lymph nodes. The left gastric artery and vein are transected at their origin. Next, the right gastroepiploic artery and the right gastric artery are transected at their origin. The duodenum is divided at least 1 cm distal to the pyloric sphincter by means of an endostapler. Subsequently, the distal esophagus is dissected from the left and right crus and mobilized, after which the distal esophagus is transected with an endostapler. Frozen section histology is performed to assess the extent of tumor invasion at the resection planes when indicated. The greater omentum is resected separately or en-bloc and marked uniformly. In the laparoscopic procedure the removal of the resected specimen with en-bloc lymphadenectomy and the greater omentum occurs via a mini-laparotomy (max. 5–6 cm), which must be muscle sparing. Next, an esophago-jejunostomy is performed by means of a Roux-en-Y reconstruction. The formation of a jejunal pouch and a feeding jejunostomy is optional [16, 17].

### Distal gastrectomy

The conventional open distal gastrectomy is performed by means of a midline laparotomy. In case of the laparoscopic procedure, the number and placement of the camera, working and assistance ports will be performed according to the surgeons’ preference. In both procedures, the lesser omentum is opened. Next, the greater curvature of the stomach is prepared. The left gastric artery and vein are transected at their origin. The gastrocolic ligament is divided at 3 cm distal to the gastroepiploic artery, after which the greater curvature is skeletonized up to the gastrosplenic ligament. The right gastroepiploic vein and artery are transected at its origin. Next the right gastric vessels are transected. The duodenum is divided distal to the pyloric sphincter by means of an endostapler. The proximal side of the stomach is divided at least 6 cm cranially from the tumor. Frozen section histology is performed to assess the extent of tumor invasion at the distal resection plane. Resection of the greater omentum is performed separately or en-bloc and marked uniformly. In the laparoscopic procedure, the removal of the resected specimen with en-bloc lymphadenectomy and omentum occurs via a mini-laparotomy (max. 5–6 cm), which must be muscle sparing. Finally, a gastro-jejunostomy is performed with Roux-en-Y reconstruction [18, 19].

### Lymphadenectomy

**Fig. 2:**
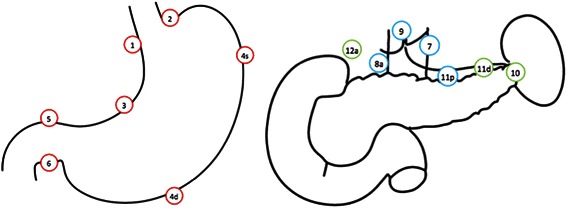
Gastric lymph nodes

Lymph node dissection is performed according to the *Dutch oncologic guidelines* and *Japanese gastric cancer treatment guidelines* [5, 20, 21]. For D2 lymphadenectomy no pancreatico-splenectomy is performed since this is associated with high postoperative morbidity and mortality without proven benefit [2]. Furthermore, lymph node station ten is not dissected during total gastrectomy since it has no additive oncological value and is associated with morbidity [2, 20]. Lymph node stations 1–3, 4d, 4sa, 4sb, 5–9, 11p, 11d and 12a are dissected during total gastrectomy. Lymph node stations 1, 3, 4d, 4sb, 5–9, 11p and 12a are dissected during distal gastrectomy (Fig. 2).

### Surgical and pathological quality control

All procedures will be carried out in one of the 10 participating centers. To ensure quality and minimize differences between the laparoscopic procedures, all surgeons have participated in the course “One day course on minimally invasive gastrectomy”, which is organized by the UMC Utrecht. All surgeons completed their learning curves (n ≥ 20) for laparoscopic gastrectomy. The centers can start with inclusion after one of the proctors (RvH or JR) has supervised at least two laparoscopic procedures on site or has reviewed at least two videos of laparoscopic gastrectomy in which predefined standards for laparoscopic gastrectomy have been met [22]. All laparoscopic operations will be recorded on video for quality control.

To ensure pathological quality, the surgical team will separately mark the resected specimen for the location of N1 lymph node stations and resection planes. The N2 lymph node will be dissected and provided in separate containers. Pathological evaluation will be performed by an experienced pathologist in each center and will be reviewed by a central pathologist in the UMC Utrecht. Pathological evaluation will be performed using a standard protocol and a checklist, developed by a central coordinating pathologist from the UMC Utrecht and including tumor characteristics, radicality, number and location of lymph nodes harvested.

### Postoperative treatment

To ensure fast recovery, the Enhanced Recovery After Surgery (ERAS) Society protocol is followed [16]. Mobilization under supervision starts immediately. On postoperative day 1, liquid oral feeding can be initiated. The postoperative treatment does not differ between both treatment arms, except for epidural analgesia, which can be initiated after open procedures. Besides an epidural, other analgesia can be given according to the local hospital’s preference and will be registered.

### Outcome measurements

Laparoscopic gastrectomy is expected to be equivalent to open gastrectomy in terms of short-term oncologic outcomes, such as R0-resection rate and number of lymph nodes harvested, but to result in less surgical trauma. The primary outcome of this study is length of postoperative hospital stay. Criteria for discharge are those for functional recovery and include: started with mobilization, oral or enteral intake according to nutritional demand, without supplementary intravenous fluids and adequate pain control with oral medication.

Secondary outcome measurements include postoperative morbidity and mortality, readmissions, cost-effectiveness, oncologic outcome and quality of life. Standardized definitions will be used for complications and include anastomotic leakage, anastomotic stricture and number of dilatations, respiratory complications, cardiac complications, chyle leakage, intra-abdominal bleeding, intra-abdominal abscess and wound infection [23]. All complications will be classified according to the Clavien-Dindo system [24]. Oncologic outcome will be measured by R0 resection rate and the number of lymph nodes resected. The validated quality of life questionnaires Euro Quality of Life-5D-5 L (EQ-5D-5 L), European Organization for Research and Treatment of Cancer Quality of Life Questionnaire 30 (EORTC QLQ-30) and the Stomach 22 module (EORTC QLQ-STO22) will be filled in pre-operative and postoperative at 6 weeks, 6, 12, 24, 36, 48 and 60 months after surgery [25–27]. Costs will be based on the recorded volumes and unit costs associated with both procedures, including costs of hospital and ICU stay, costs of operating rooms and costs associated with complications and reoperations. Effect will be based on the quality of life and productivity of patients. Productivity will be measured with the Short Form Health and Labour Questionnaire (SF-HLQ) [28].

Other study parameters include baseline characteristics (gender, age, American Society of Anesthesiologists (ASA) classifications, BMI, comorbidities, perioperative chemotherapy), peri-operative outcomes (blood loss, duration of surgery, conversion rate), survival (overall and disease free), patients experience (Visual Analogue Scale ;VAS) for pain, time to return to normal nutrition regime and time to return to daily activity) and weight. Surgeons’ ergonomics are measured by means of the Subjective Mental Effort Questionnaire (SMEQ) [29].

### Sample size calculation

The primary outcome parameter is length of postoperative hospital stay. The hypothesis is that laparoscopic gastrectomy will result in a shorter postoperative hospital stay compared with open gastrectomy. A recent meta-analysis showed that a laparoscopic procedure shortened the median hospital stay from 18 to 14 days [7]. It was calculated (α = 0.05, Power = 0.80) that 210 patients (105 in each treatment arm) are required to detect this 4-day reduction in postoperative hospital stay.

### Statistical analysis

Analysis will take place using SPSS statistical software (SPSS Inc. Chicago, Illinois, USA) and R statistical computing (R Foundation for Statistical Computing, Vienna, Austria). Data analysis will be performed on an intention-to-treat basis. Additional per-protocol analysis will be performed for tumor type, tumor stage and type of gastrectomy (distal versus total).

Differences in improvement in primary and secondary outcomes between interventions are analyzed using linear mixed-effects modeling or longitudinal Poisson regression, taking relevant patient characteristics into account [30]. Missing values will be imputed using multiple imputation techniques. Kaplan-Meier survival curves will be computed to evaluate differences in disease-free and overall survival. Log-rank tests will be used to compare survival curves and the Cox regression model will be used to accomplish multivariate analysis.

Cost-effectiveness will be calculated by comparing costs and effects in relation to both strategies up until one year after the operation. A societal perspective will be used for this analysis, i.e. medical and non-medical direct and indirect costs will be taken into account. After analyzing mean costs and effects for both strategies an incremental cost-effectiveness ratio will be calculated. Results will be presented using incremental cost-effectiveness planes and cost-effectiveness acceptability curves. Costs and effects will be discounted according to Dutch guidelines. Bootstrapping will be used to assess uncertainty in the balance between costs and effects.

### Interim analysis

Outcomes will be evaluated by the DSMB after 105 patients are included, using the Peto approach (p < 0.001). Stop-criteria are: <70 % R0-resections in one of the study arms and <50 % of 10 harvested lymph nodes in one of the study arms. If the DSMB suspects any adverse effects, a meeting will be organized between the DSMB, the trial research group and an independent statistician. The final decision is made by the DSMB. Their opinion is sent to the study coordinator and principal investigator. A copy of their advice will be sent to the ethics committee. The trial will not be stopped for futility (no difference in postoperative hospital stay between surgical procedures) as the outcome of all endpoints of this randomized controlled trial on this subject are relevant to healthcare professionals involved with these procedures in Western hospitals.

## Discussion

The LOGICA-trial is a non-blinded, multicenter, prospectively randomized controlled trial, comparing laparoscopic versus open gastrectomy, which is the gold standard in patients with resectable gastric adenocarcinoma. In the revised 2010 Japanese gastric cancer treatment guidelines, open gastrectomy is considered the first-choice procedure for patients with resectable gastric carcinoma [5]. However, this procedure is associated with considerable morbidity [6].

Minimally invasive techniques have shown to improve perioperative outcomes in other procedures such as colectomy for colonic cancer and esophagectomy for esophageal carcinoma [31, 32]. For gastrectomy, studies from Asian populations have shown a benefit for the patient after laparoscopic gastrectomy compared to open gastrectomy [7, 8, 10–12]. Laparoscopic gastrectomy was associated with lower intraoperative blood loss, reduced risk of postoperative complications and shorter hospital stay. Resection margin, lymph node retrieval and 5-year survival rate were comparable. This was at the cost of longer operative time [7, 8, 10–12]. However, Western populations have a more advanced stage tumor, that is located more frequently in the proximal stomach and diagnosed more often at an older age compared with the Asian population. Therefore, it is unknown whether the results of these studies can be extrapolated to the Western population [13, 14]. Furthermore, this trial can be used to evaluate the laparoscopic techniques used.

In the last decennium, laparoscopic gastrectomy has been introduced in several centers in the Netherlands. To evaluate this technique, a randomized trial is needed.

## Conclusion

This is a randomized controlled trial comparing laparoscopic gastrectomy with the gold standard open gastrectomy for surgically resectable gastric carcinoma in a Western population. It is hypothesized that laparoscopic gastrectomy will result in a shorter postoperative hospital stay, lower postoperative morbidity, less readmissions, higher cost-effectiveness, better postoperative quality of life, with similar mortality and oncologic outcomes, compared to open gastrectomy.

### Trial status

The independent ethics committee of the UMC Utrecht (NL47444.041.14) approved the trial protocol. Recruitment of patients started in December 2014.

## Abbreviations

DSMB: Data Safety Monitoring Board
ECOG: European Clinical Oncology Group
UMC Utrecht: University Medical Center Utrecht
EQ-5D-5L: Euro Quality of Life-5D-5L
EORTC: European Organisation for Research and Treatment of Cancer
SMEQ: Subjective Mental Effort Questionnaire
SF-HLQ: Short Form Health and Labour Questionnaire
ASA: American Society of Anesthesiologists
CRP: C-reactive protein
BMI: Body Mass Index
VAS: Visual Analogue Scale
TNM: Tumor Node Metastasis

## References

[1] Ferlay J, Soerjomataram I, Dikshit R, Eser S, Mathers C, Rebelo M, et al. Cancer incidence and mortality worldwide: sources, methods and major patterns in GLOBOCAN 2012. Int J Cancer 2015 Mar 1;136(5):E359-86. 10.1002/ijc.29210 25220842

[2] Hartgrink HH, van de Velde CJ, Putter H, Bonenkamp JJ, Klein Kranenbarg E, Songun I (2004). Extended lymph node dissection for gastric cancer: who may benefit? Final results of the randomized Dutch gastric cancer group trial. J Clin Oncol.

[3] Cunningham D, Allum WH, Stenning SP, Thompson JN, Van de Velde CJ, Nicolson M (2006). Perioperative chemotherapy versus surgery alone for resectable gastroesophageal cancer. N Engl J Med.

[4] Ronellenfitsch U, Schwarzbach M, Hofheinz R, Kienle P, Kieser M, Slanger TE (2013). Perioperative chemo(radio)therapy versus primary surgery for resectable adenocarcinoma of the stomach, gastroesophageal junction, and lower esophagus. Cochrane Database Syst Rev.

[5] Association JGC (2011). Japanese gastric cancer treatment guidelines 2010 (ver. 3). Gastric Cancer.

[6] Memon MA, Subramanya MS, Khan S, Hossain MB, Osland E, Memon B (2011). Meta-analysis of D1 versus D2 gastrectomy for gastric adenocarcinoma. Ann Surg.

[7] Haverkamp L, Weijs TJ, van der Sluis PC, van der Tweel I, Ruurda JP, van Hillegersberg R (2013). Laparoscopic total gastrectomy versus open total gastrectomy for cancer: a systematic review and meta-analysis. Surg Endosc.

[8] Zeng YK, Yang ZL, Peng JS, Lin HS, Cai L (2012). Laparoscopy-assisted versus open distal gastrectomy for early gastric cancer: evidence from randomized and nonrandomized clinical trials. Ann Surg.

[9] Kitano S, Iso Y, Moriyama M, Sugimachi K (1994). Laparoscopy-assisted Billroth I gastrectomy. Surg Laparosc Endosc.

[10] Xiong JJ, Nunes QM, Huang W, Tan CL, Ke NW, Xie SM (2013). Laparoscopic vs open total gastrectomy for gastric cancer: a meta-analysis. World J Gastroenterol.

[11] Wang W, Zhang X, Shen C, Zhi X, Wang B, Xu Z (2014). Laparoscopic versus open total gastrectomy for gastric cancer: an updated meta-analysis. PLoS ONE.

[12] Vinuela EF, Gonen M, Brennan MF, Coit DG, Strong VE (2012). Laparoscopic versus open distal gastrectomy for gastric cancer: a meta-analysis of randomized controlled trials and high-quality nonrandomized studies. Ann Surg.

[13] Dicken BJ, Bigam DL, Cass C, Mackey JR, Joy AA, Hamilton SM (2005). Gastric adenocarcinoma: review and considerations for future directions. Ann Surg.

[14] Griffin SM (2005). Gastric cancer in the East: same disease, different patient. Br J Surg.

[15] Vereniging Integrale Kankercentra. Diagnostiek, behandeling en follow-up van het maagcarcinoom [Internet]. 2009. Available from: http://www.oncoline.nl/uploaded/docs/Maagcarcinoom/Richtlijn%20maagcarcinoom.pdf.

[16] Mortensen K, Nilsson M, Slim K, Schafer M, Mariette C, Braga M, et al. Consensus guidelines for enhanced recovery after gastrectomy: Enhanced Recovery After Surgery (ERAS(R)) Society recommendations. Br J Surg. 2014;21. 10.1002/bjs.9582 25047143

[17] Fein M, Fuchs KH, Thalheimer A, Freys SM, Heimbucher J, Thiede A (2008). Long-term benefits of Roux-en-Y pouch reconstruction after total gastrectomy: a randomized trial. Ann Surg.

[18] Zong L, Chen P (2011). Billroth I vs. Billroth II vs. Roux-en-Y following distal gastrectomy: a meta-analysis based on 15 studies. Hepatogastroenterology.

[19] Shim JH, Oh SI, Yoo HM, Jeon HM, Park CH, Song KY. Roux-en-Y Gastrojejunostomy After Totally Laparoscopic Distal Gastrectomy: Comparison With Billorth II Reconstruction. Surg Laparosc Endosc Percutan Tech. 2014;4. 10.1097/SLE.0b013e31829014ea 24710243

[20] Oncoline. Landelijke werkgroep Gastro-intestinale Tumoren Versie: 1.0. [Internet].; 2009. Available from: http://oncoline.nl/maagcarcinoom.

[21] Lee JH, Lee HJ, Kong SH, Park do J, Lee HS, Kim WH (2014). Analysis of the lymphatic stream to predict sentinel nodes in gastric cancer patients. Ann Surg Oncol.

[22] Markar SR, Wiggins T, Ni M, Steyerberg EW, Van Lanschot JJ, Sasako M (2015). Assessment of the quality of surgery within randomised controlled trials for the treatment of gastro-oesophageal cancer: a systematic review. Lancet Oncol.

[23] Low DE, Alderson D, Cecconello I, Chang AC, Darling GE, D’Journo XB, et al. International Consensus on Standardization of Data Collection for Complications Associated With Esophagectomy: Esophagectomy Complications Consensus Group (ECCG). Ann Surg. 2015;20. 10.1097/SLA.0000000000001098 25607756

[24] Dindo D, Demartines N, Clavien PA (2004). Classification of surgical complications: a new proposal with evaluation in a cohort of 6336 patients and results of a survey. Ann Surg.

[25] Aaronson NK, Ahmedzai S, Bergman B, Bullinger M, Cull A, Duez NJ (1993). The European Organization for Research and Treatment of Cancer QLQ-C30: a quality-of-life instrument for use in international clinical trials in oncology. J Natl Cancer Inst.

[26] Blazeby JM, Conroy T, Bottomley A, Vickery C, Arraras J, Sezer O (2004). Clinical and psychometric validation of a questionnaire module, the EORTC QLQ-STO 22, to assess quality of life in patients with gastric cancer. Eur J Cancer.

[27] Herdman M, Gudex C, Lloyd A, Janssen M, Kind P, Parkin D, et al. Development and preliminary testing of the new five-level version of EQ-5D (EQ-5D-5 L). Qual Life Res. 2011;20(10):1727–36. 10.1007/s11136-011-9903-x PMC3220807 21479777

[28] van Roijen L, Essink-Bot ML, Koopmanschap MA, Bonsel G, Rutten FF (1996). Labor and health status in economic evaluation of health care. The Health and Labor Questionnaire. Int J Technol Assess Health Care.

[29] Zijlstra FRH, Doorn L (1985). The construction of a scale to measure subjective effort. Technical Report, Delft University of Technology, Department of Philosophy and Social Sciences.

[30] Jos W. R. Twisk. Applied Longitudinal Data Analysis for Epidemiology A Practical Guide. 2nd ed. Cambridge: Cambridge University Press; 2013.

[31] van der Pas MH, Haglind E, Cuesta MA, Furst A, Lacy AM, Hop WC (2013). Laparoscopic versus open surgery for rectal cancer (COLOR II): short-term outcomes of a randomised, phase 3 trial. Lancet Oncol.

[32] Biere SS, van Berge Henegouwen MI, Maas KW, Bonavina L, Rosman C, Garcia JR (2012). Minimally invasive versus open oesophagectomy for patients with oesophageal cancer: a multicentre, open-label, randomised controlled trial. Lancet.

